# Bilateral Vocal Cord Paralysis and Cervicolumbar Radiculopathy as the Presenting Paraneoplastic Manifestations of Small Cell Lung Cancer: A Case Report and Literature Review

**DOI:** 10.1155/2016/2868190

**Published:** 2016-09-07

**Authors:** Jeffrey C. Yeung, C. Elizabeth Pringle, Harmanjatinder S. Sekhon, Shaun J. Kilty, Kristian Macdonald

**Affiliations:** ^1^Department of Otolaryngology-Head & Neck Surgery, University of Ottawa, Ottawa, ON, Canada; ^2^Division of Neurology, Department of Medicine, University of Ottawa, Ottawa, ON, Canada; ^3^Department of Pathology & Laboratory Medicine, University of Ottawa, Ottawa, ON, Canada

## Abstract

*Introduction*. Bilateral vocal cord paralysis (BVCP) is a potential medical emergency. The Otolaryngologist plays a crucial role in the diagnosis and management of BVCP and must consider a broad differential diagnosis. We present a rare case of BVCP secondary to anti-Hu paraneoplastic syndrome.* Case Presentation*. A 58-year-old female presented to an Otolaryngology clinic with a history of progressive hoarseness and dysphagia. Flexible nasolaryngoscopy demonstrated BVCP. Cross-sectional imaging of the brain and vagus nerves was negative. An antiparaneoplastic antibody panel was positive for anti-Hu antibodies. This led to an endobronchial biopsy of a paratracheal lymph node, which confirmed the diagnosis of small cell lung cancer.* Conclusion*. Paraneoplastic neuropathy is a rare cause of BVCP and should be considered when more common pathologies are ruled out. This is the second reported case of BVCP as a presenting symptom of paraneoplastic syndrome secondary to small cell lung cancer.

## 1. Introduction

Bilateral vocal cord paralysis (BVCP) is a potentially life-threatening emergency and the presenting symptoms include upper airway obstruction, dyspnea, and stridor. The Otolaryngologist-Head and Neck Surgeon has an integral role in the evaluation, diagnosis, and management of patients with such symptoms. In adults, the most common etiologies of BVCP include iatrogenic injury, direct tumour compression, neurologic disease, and idiopathic etiologies [1]. In the event that imaging of the brain and recurrent laryngeal nerve is normal, the Otolaryngologist must broaden his/her differential diagnosis and consider more infrequent causes before concluding that BVCP is idiopathic. We present a rare case of BVCP secondary to anti-Hu paraneoplastic syndrome.

## 2. Case Report

A 58-year-old female was referred to an Otolaryngology outpatient clinic with a 6-month history of progressive hoarseness and dysphagia, associated with a 20-pound weight loss.

On further history examination, she had several seemingly unassociated medical symptoms over the previous year, including right hand and lower limb pain and weakness. She was subsequently diagnosed with carpal tunnel syndrome and right C6, left C7, and right L5 radiculopathy.

Four months prior to the current presentation, she was diagnosed with right middle lobe pneumonia and treated with a course of antibiotics. Despite medical management, she had persistent symptoms and consequently a computed tomography (CT) scan of the thorax was ordered. This scan demonstrated right middle lobe consolidation and atelectasis with mediastinal and hilar lymphadenopathy. Flexible bronchoscopy was performed at the time, and bronchoalveolar lavage of the right middle lobe, brush biopsies, and endobronchial biopsies were negative for malignancy. At that time, her vocal cords were documented to be normal. A follow-up CT scan demonstrated moderate resolution of the pneumonia.

The patient's relevant past medical history included a 40-pack-year smoking history and chronic obstructive pulmonary disease. She had no known medication allergies. There was no previous history of head and neck surgery.

On physical examination, she was cachectic in appearance. She had audible inspiratory stridor and increased work of breathing. Flexible nasolaryngoscopy revealed bilateral vocal cord paralysis. There was no palpable neck mass. The remainder of the cranial nerve examination was normal, as was the remainder of the head and neck examination.

Informed consent was obtained and the patient was taken to the operating room for a tracheostomy. Postoperatively, a CT scan of the skull base to the aortic arch was ordered and no lesion along the course of either vagus nerve was found. A modified barium swallow demonstrated mild oral and moderate pharyngeal phase dysphagia with frank aspiration, and, as a result, enteral feeding was initiated.

**Figure 1:**
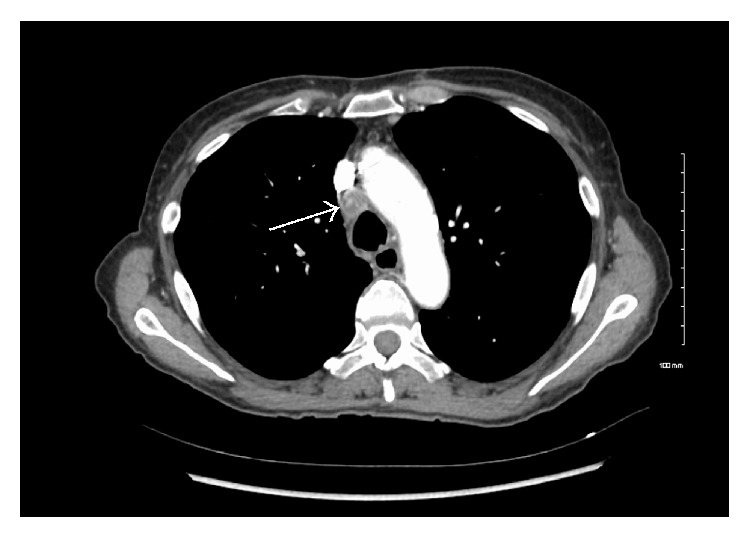
CT scan of the thorax demonstrating necrotic paratracheal node. Enhanced axial CT scan of the thorax, mediastinal window, in a 58-year-old female who presented with bilateral vocal cord immobility of unknown etiology. The positive serum anti-Hu antibody, which is highly associated with small cell lung carcinoma, led to this repeat CT scan. The arrow demonstrates an enlarged level 4R paratracheal lymph node with central necrosis. See Figure 2 for the pathologic description of a biopsy from this node.

**Figure 2:**
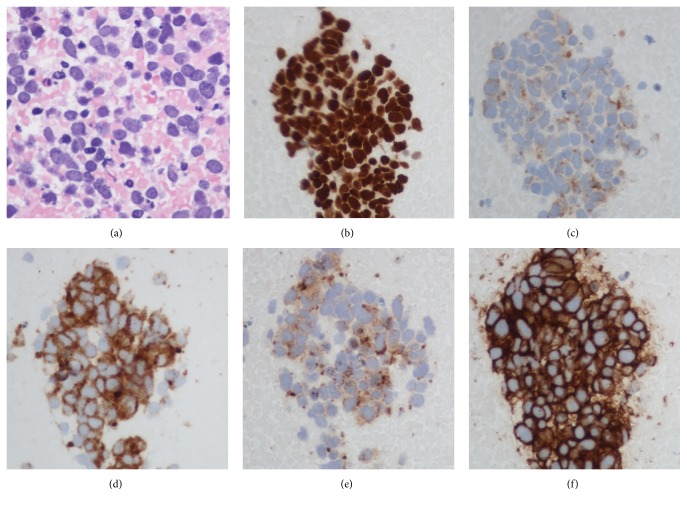
Representative cytology slides of paratracheal lymph node. Cytology of endobronchial ultrasound guided fine needle biopsy from the necrotic mediastinal lymph node in Figure 1. Hematoxylin and eosin stain of cell block section (a) demonstrated crowded, overlapping groups of malignant cells in a background of necrosis. Immunohistochemistry was positive for TTF-1 (b), AE-1/AE-3, perinuclear dot-like positivity (c), synaptophysin (d), chromogranin (e), and CD56 (f), confirming the diagnosis of small cell lung carcinoma.

The patient went on to develop polyradiculoneuropathy, bilateral facial weakness, and recurrent tachyarrhythmias. Electrophysiologic studies were repeated and revealed length-dependent axonal sensorimotor polyneuropathy, progressive compared to her previous study. The study did not demonstrate hallmarks of acute inflammatory demyelinating polyradiculoneuropathy (namely, conduction block or temporal dispersion). Pending further investigations, a trial of intravenous immunoglobulin was initiated. A full workup, including magnetic resonance imaging of the brain, lumbar puncture, vasculitic markers, and serum/urine protein electrophoresis, was ordered. Cerebrospinal fluid analysis demonstrated mild pleiocytosis and elevated protein levels but no malignant cells. An anti-paraneoplastic antibody panel was positive for anti-Hu antibodies. A repeat CT scan of the thorax was performed, which demonstrated a new necrotic level 4R paratracheal lymph node (Figure 1). An endobronchial ultrasound guided biopsy of this node confirmed the diagnosis of small cell lung cancer (SCLC) (Figure 2). A complete oncologic workup was subsequently performed and no primary tumour or distant metastases were identified.

The patient's symptoms did not respond to therapeutic trials of intravenous immunoglobulin, plasmapheresis, or chemotherapy (carboplatin, etoposide, and dexamethasone). She unfortunately developed febrile neutropenia and acute hypoxemic respiratory failure and was admitted to the intensive care unit for positive pressure ventilation. Her condition continued to worsen and she unfortunately passed away following withdrawal of ventilatory support.

## 3. Discussion

We used the search query (“vocal cord paralysis” OR (“vocal” AND “cord” AND “paralysis”) OR “vocal cord paralysis”) AND (anti-Hu OR paraneoplastic) in Pubmed until October 1st, 2015. We identified one other reported case of bilateral vocal cord paralysis as a paraneoplastic manifestation of SCLC [2, 3]. Similar to the patient described in our case report, this patient had also developed several somatic complaints before BVCP was diagnosed, including psychiatric symptoms, paresthesias, vertigo, anxiety, and depression. The patient had also recently been diagnosed with fibromyalgia and systemic lupus erythematosus. Years after these initial symptoms manifested, she was referred to an Otolaryngologist for progressive weight loss, dysphagia, and dysphonia and was then diagnosed with BVCP.

### 3.1. Paraneoplastic Neuropathy

Several malignancies have a higher propensity to cause paraneoplastic neuropathies, including SCLC, lymphoma, adenocarcinoma, and thymic carcinoma [4]. Paraneoplastic neuropathy associated with anti-Hu antibodies (or anti-Hu syndrome) commonly presents as encephalomyelitis, sensory neuronopathy, cerebellar degeneration, and autonomic neuropathy [5]. The diagnosis can typically be confirmed by the presence of autoantibodies in serum. In the case of anti-Hu syndrome, the majority of patients with paraneoplastic sensory neuronopathy are seropositive, though up to 16% of patients can be seronegative [4].

### 3.2. Hu Antigens and Anti-Hu Antibodies

The Hu antigens are a family of intranuclear and intracytoplasmic proteins that are expressed by all neurons of central and peripheral nervous systems. These antigens are also present in almost all SCLC tumour cells but are characteristically absent in most normal nonneuronal cells [6]. The function of these proteins is not currently known, but it is postulated that they promote differentiation and maintenance of the neuronal phenotype. The anti-Hu IgG antibodies were discovered in 1985, identified in the CSF of a patient with SCLC [7]. The antibody and antigen now bear the name of this index patient.

The presence of anti-Hu antibodies in serum carries a 99% specificity for SCLC [8]. Consequently, the prospect of anti-Hu antibodies as an early marker and prognostic indicator for SCLC has been proposed [9]. However, anti-Hu antibodies have also been identified in patients with various other malignancies such as lymphoma, thymic carcinoma, neuroblastoma, synovial carcinoma, nonseminomatous testicular germ cell tumours, and carcinoma of the gallbladder [6, 10–14]. Anti-Hu antibodies have also been identified in pediatric patients with autoimmune nonparaneoplastic limbic encephalitis [15].

### 3.3. Clinical Manifestations, Diagnostic Criteria, and Management of Anti-Hu Syndrome

In addition to the more common manifestations listed above, the most common cranial nerve manifestation in patients with anti-Hu syndrome is subacute sensorineural hearing loss [16, 17]. In anti-Hu syndrome, neurological symptoms typically precede tumour detection by a median of 7.5 months (range: 3–31 months) [18]. The neurological deficits associated with anti-Hu syndrome are typically progressive.

The presence of serum anti-Hu antibodies has been associated with improved prognosis in patients with underlying malignancies. In a series of 196 patients with SCLC, patients positive for anti-Hu antibodies (*n* = 32) demonstrated improved response to chemotherapy (55.6% versus 19.6%) and improved survival (14.9 versus 10.2 months), compared to those who were negative for anti-Hu antibodies [19]. It is hypothesized that the patients who produce anti-Hu antibodies are able to mount an immune response against the tumour, thus conferring them with a better prognosis. This was further alluded to in a case of spontaneous tumour regression seen in the setting of anti-Hu syndrome and SCLC [20].

Diagnostic criteria for paraneoplastic neuropathies were previously described and are divided into “definite” and “possible” based on the presence or absence of classical paraneoplastic phenomena and/or onconeural antibodies [21]. While our patient did not present with a classical paraneoplastic syndrome, she was found to have onconeural antibodies in her serum and would therefore meet criteria for definite paraneoplastic syndrome, as defined by Graus and Dalmau. The diagnosis of anti-Hu paraneoplastic syndrome requires 3 criteria: (1) clinical signs of central or peripheral neuropathy, (2) no direct tumour infiltration, compression, or metastasis to the nervous system, and (3) presence of serum anti-Hu antibodies [4]. Other systemic autoantibodies (such as anti-DNA, anti-centromere, anti-Ro, and anti-La) are present in up to 33% of patients with anti-Hu syndrome, and this may potentially confound the diagnosis. The diagnosis of anti-Hu syndrome should be followed by investigations to rule out an underlying malignancy, specifically SCLC.

The management of anti-Hu syndrome should be directed at identifying and treating the underlying malignancy, as well as rehabilitation of the neurological deficits. Immunosuppressants and immunomodulators previously described for use in anti-Hu syndrome include corticosteroids, rituximab, plasmapheresis, and intravenous immunoglobulins [4]. The outcomes of these therapies have not been studied extensively in large sample sizes, as documented by a recent Cochrane review [22]. The current body of literature consists only of small case series, case reports, and expert opinion. A recent example, an open-label study of sirolimus, did not find a significant improvement compared to other immunotherapies, with only 2 out of 17 patients demonstrating a response [23]. Currently, there is a lack of evidence endorsing the use of immunosuppressants and immunomodulators in anti-Hu syndrome.

## 4. Conclusion

We describe the second reported case of bilateral vocal cord immobility associated with anti-Hu paraneoplastic syndrome and small cell lung carcinoma. After ruling out more common etiologies, it may be helpful for the clinician to consider this diagnosis when investigating patients with bilateral vocal cord paralysis.
